# A case in which laparoscopic right salpingo-oophorectomy was performed in a patient with a lumboperitoneal shunt

**DOI:** 10.1016/j.ijscr.2020.05.065

**Published:** 2020-06-06

**Authors:** Mio Nakagawa, Tomomi Egawa-Takata, Yuri Kamino, Ayuko Otoshi, Yayoi Fukuda, Yoshimi Tokugawa, Chikako Tsukahara, Takashi Miyatake, Yukihiro Nishio

**Affiliations:** Department of Obstetrics and Gynecology, Osaka Police Hospital, 10-31 Kitayama-cho Tennoji-ku, Osaka 543-0035, Japan

**Keywords:** LP shunt, lumboperitoneal shunt, VP shunt, ventriculoperitoneal shunt, CT, computed tomography, MRI, magnetic resonance imaging, iNPH, idiopathic normal pressure hydrocephalus, Case report, Laparoscopic surgery, Lumboperitoneal shunt, Salpingo-oophorectomy, Ventriculoperitoneal shunt, Ovarian cyst

## Abstract

**Introduction:**

Ventriculoperitoneal (VP shunts) and lumboperitoneal shunts (LP shunts) are used to treat hydrocephalus. The effectiveness of LP shunts has recently been demonstrated. Due to population aging, the number of patients with LP shunts is expected to increase.

**Presentation of case:**

A 51-year-old female, in whom an LP shunt had been inserted to treat hydrocephalus after a subarachnoid hemorrhage, underwent laparoscopic right salpingo-oophorectomy for a right endometriotic cyst. We consulted a neurosurgeon and confirmed the route of the shunt. We started a normal laparoscopic procedure. The head of the shunt tube was located in Douglas’ pouch and was an obstacle to the procedure. We moved the head of the shunt tube to the vesicouterine pouch and successfully conducted the standard operation. We report the case together with a literature review.

**Discussion:**

There have been several reports about gynecological laparoscopic surgery being performed in patients with VP shunts. On the other hand, we did not find any English literature about gynecological laparoscopic surgery being conducted in patients with LP shunts during a PubMed search.

**Conclusion:**

This is the first report about gynecological laparoscopic surgery being performed in a patient with an LP shunt.

## Highlights

• There are few reports about laparoscopic surgery involving patients with LP shunts.
• This is the first report about laparoscopic gynecological surgery with an LP shunt.
• In gynecological operations, the head of the shunt tube can be an obstacle.
• The head of the shunt tube might need to be moved for gynecological operations.

## 1 Introduction

Lumboperitoneal shunts (LP shunts) are used to treat hydrocephalus. Ventriculoperitoneal shunt (VP shunt) surgery is the most common treatment for hydrocephalus in North America and Europe. On the other hand, LP shunts are increasingly being used to treat hydrocephalus in Japan because they do not require cranial surgery, and patients are more willing to undergo lumbar surgery than cranial surgery. LP shunt surgery for idiopathic normal pressure hydrocephalus (iNPH) was recently demonstrated to be safe and effective. The prevalence of iNPH is 1.1% among elderly Japanese people and 2.1% among the elderly Swedish population. Due to population aging, the number of patients with LP shunts is expected to increase [1,2].

There have been a number of reports about laparoscopic surgery involving patients with VP shunts, and there have also been several reports about laparoscopic gynecological surgery involving patients with VP shunts. However, there have only been two reports about laparoscopic surgery involving patients with LP shunts in the English literature, and no reports about laparoscopic gynecological surgery involving patients with LP shunts were found during a PubMed search [3,4].

This is the first report about laparoscopic gynecological surgery involving a patient with an LP shunt.

Here, we report a case in which laparoscopic right salpingo-oophorectomy was performed in a patient with an LP shunt, together with a review of the literature. This work has been reported in line with the SCARE criteria [5].

## 2 Case presentation

A 51-year-old, gravida 1, para 1, Japanese female complained of abnormal genital bleeding for two months and presented to a clinic. An ovarian tumor was found during abdominal computed tomography (CT), and so the patient was referred to our hospital. The abnormal genital bleeding had stopped when she visited our hospital. An ultrasound scan of her right ovary revealed a swollen region of 7 cm in diameter, which contained multiple cysts, and the uterine endometrium was 9-mm-thick. Cervical cytology and an endometrial biopsy produced normal findings.

**Fig. 1:**
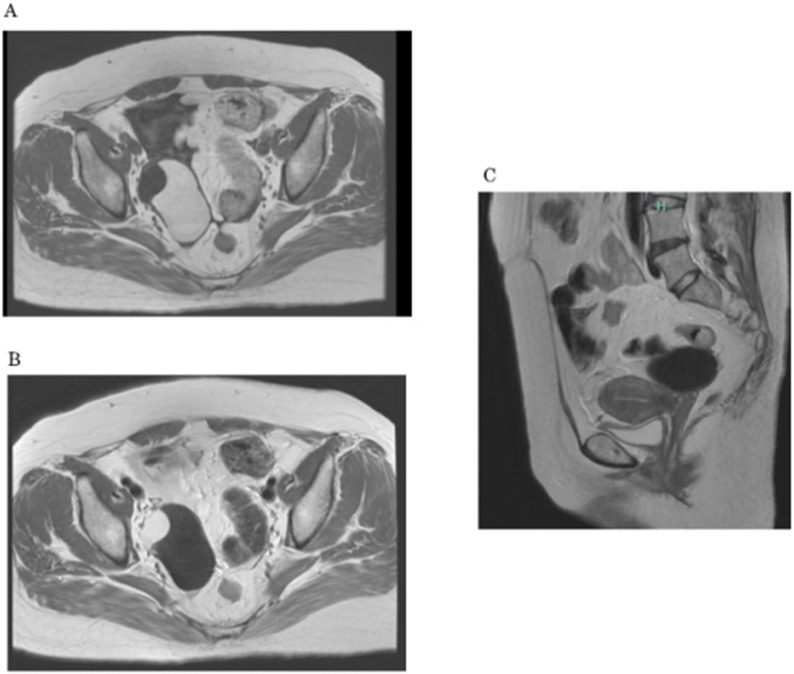
MRI images. A. T1-weighted imaging showed a 7-cm multilocular ovarian cyst, which exhibited regions of high and low signal intensity. B. A T2-weighted image is shown. C. T1-weighted sagittal imaging showed that the ovarian cyst was located behind the uterus.

**Fig. 2:**
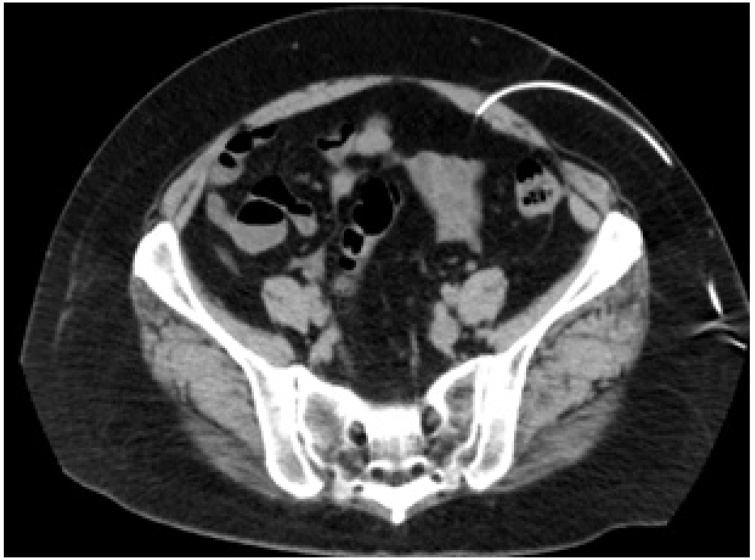
CT image of the route of the shunt. The shunt tube ran through the left flank to the abdominal cavity.

**Fig. 3:**
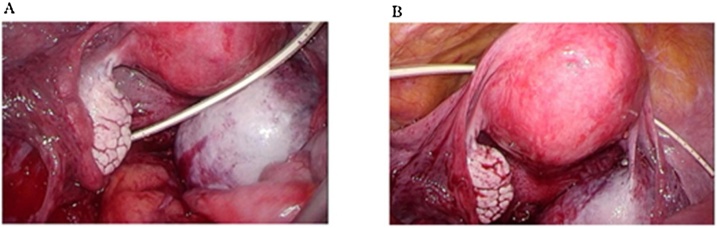
Photos of the shunt tube obtained during the operation. A. The head of the shunt tube was located in Douglas’ pouch. B. The head of the shunt tube was moved to the vesicouterine pouch.

On magnetic resonance imaging (MRI), an ovarian tumor, which measured 7 cm in diameter and contained multiple cysts, was detected, and a large part of the tumor exhibited high signal intensity on T1-weighted imaging and low signal intensity on T2-weighted imaging. No solid components were detected (Fig. 1). We decided to perform a laparoscopic right salpingo-oophorectomy. The patient’s medical history included endometriosis from the age of 25 without specific therapy and subarachnoid hemorrhaging due to the rupturing of an aneurysm at the age of 43. The patient was diagnosed with hydrocephalus after she underwent surgery for the subarachnoid hemorrhaging, and an LP shunt was inserted. Her medical history also included kidney stones, schizophrenia, hypertension, and diabetes mellitus at the age of 50. We confirmed the route of the LP shunt on a CT scan, which had been conducted at another clinic. It revealed that the LP shunt had been placed from her left flank to Douglas’ pouch (Fig. 2). Under general anesthesia, laparoscopic right adnexectomy was performed. A 12-mm trocar was inserted at the umbilicus, and three 5-mm trocars were inserted 3 cm inside the right and left upper anterior iliac crests and on the midline of the lower abdomen. The abdominal pressure was set at 8 mmHg. The ovarian tumor was located in Douglas’ pouch and had adhered to the back of the uterus. Also, the head of the shunt tube was located in Douglas’ pouch and was an obstacle to the operation. We temporarily shifted the head of the shunt tube from Douglas’ pouch to the vesicouterine pouch to prevent damage to the shunt and ensure that the operation could be conducted smoothly (Fig. 3). The operation time was 2 h and 11 min, and the total volume of intraoperative blood loss was 50 ml. The patient’s postoperative course was uneventful, and she was discharged on postoperative day 3. The histological diagnosis was an endometriotic cyst. The patient was examined at 1 month after the surgery at our hospital’s outpatient clinic, and no adverse events were observed. She was followed-up at the outpatient clinic of a general practitioner.

## 3 Discussion

In cases in which patients with peritoneal shunts undergo surgery, various management strategies, such as shunt clamping and catheter removal, have been reported to prevent possible adverse events, such as shunt infection, pneumocephalus, and shunt obstruction. Torigoe et al. reported that they clamped a shunt catheter with atraumatic forceps in the subcutaneous region under radiographic guidance before conducting laparoscopic colectomy in a patient with a VP shunt [6]. On the other hand, in recent years, there have been several reports about operations that were performed safely in patients with peritoneal shunts without any particular management strategies being employed because cerebrospinal fluid shunts have unidirectional valves, which prevent backflow. It is assumed that most of the LP shunts used since the 1990s had unidirectional valves. In addition, the laparoscopic insertion of a cerebrospinal fluid shunt itself was recently reported, and it was suggested that this technique is associated with a better prognosis than mini-laparotomy [7]. However, Raskin et al. reported a case in which pneumocephalus occurred after a laparoscopic bilateral salpingo-oophorectomy, involving abdominal pressurization to 50 mmHg, in a patient with a VP shunt. In the latter case, the shunt had been inserted more than 20 years ago. It is conceivable that the tube was old and allowed backflow through the valve. Also, an abdominal pressure of 50 mmHg is considered to be too high although Neales et al. reported that no reflux through shunt tubes occurred at a pressure of 80 mmHg, based on an in vitro experiment [8,9]. Matsumoto also reported that no reflux occurred through shunt tubes at a pressure of 25 mmHg [10]. Our procedure was performed at an abdominal pressure level of 8 mmHg, which safely prevented adverse events.

In the current case, we needed to move the head of the shunt tube from Douglas’ pouch to the vesicouterine pouch because it was an obstacle to the dissection of adhesions caused by endometriosis. It was not difficult to relocate the head of the shunt tube to the vesicouterine pouch; however, during hysterectomy it might be difficult to decide where to place the heads of shunt tubes because hysterectomy requires a wider operation field than salpingo-oophorectomy. Also, during hysterectomy shunt tubes can become infected with bacteria from the vagina if they are placed near the vaginal wall after the wall is cut.

Bush et al. reported a case in which robotic surgery was performed in a patient with a VP shunt, and they fixed the head of the shunt tube to the abdominal wall using a clip to ensure that the operation was conducted smoothly [11].

There have been several reports about laparoscopic surgery being conducted in patients with VP shunts. On the other hand, there have only been two reports about laparoscopic surgery being carried out in patients with LP shunts. Imagami et al. reported that they performed a standard laparoscopic operation, while paying attention to port placement, in a case involving a colorectal cancer patient with an LP shunt [4].

There is no standard management strategy for patients with peritoneal shunts who undergo laparoscopic surgery; however, most laparoscopic surgery can be performed in such patients if careful perioperative observation is employed. In gynecological operations, the head of the shunt tube can be an obstacle, and therefore, might need to be moved.

## 4 Conclusion

Laparoscopic right salpingo-oophorectomy was performed successfully in a patient with an LP shunt, without any special management techniques being required. However, it was necessary to move the head of the shunt tube slightly.

## Declaration of Competing Interest

The authors have no conflicts of interest.

## Sources of funding

None.

## Ethical approval

This is a case report and doesn’t need ethical approval.

## Consent

The patient provided unconditional informed consent to the authors to report the findings.

## Author contribution

Mio Nakagawa and Tomomi Egawa-Takata wrote the paper.

Yuri Kamino, Ayuko Otoshi, Yayoi Fukuda, Yoshimi Tokugawa, Chikako Tsukahara, Takashi Miyatake, Yukihiro Nishio were involved.

## Registration of research studies

researchregistry5559.

## Guarantor

Tomomi Egawa-Takata.

## Provenance and peer review

Not commissioned, externally peer-reviewed.
